# Leukocyte Inclusion within a Platelet Rich Plasma-Derived Fibrin Scaffold Stimulates a More Pro-Inflammatory Environment and Alters Fibrin Properties

**DOI:** 10.1371/journal.pone.0121713

**Published:** 2015-03-30

**Authors:** Eduardo Anitua, Mar Zalduendo, María Troya, Sabino Padilla, Gorka Orive

**Affiliations:** 1 Foundation Eduardo Anitua, Vitoria, Spain; 2 Laboratory of Regenerative Medicine, Biotechnology Institute (BTI), Vitoria, Spain; Maastricht University Faculty of Health, Medicine, and Life Sciences, NETHERLANDS

## Abstract

One of the main differences among platelet-rich plasma (PRP) products is the inclusion of leukocytes that may affect the biological efficacy of these autologous preparations. The purpose of this study was to evaluate whether the addition of leukocytes modified the morphological, biomechanical and biological properties of PRP under normal and inflammatory conditions. The release of pro-inflammatory cytokines from plasma rich in growth factors (PRGF) and leukocyte-platelet rich plasma (L-PRP) scaffolds was determined by enzyme-linked immunosorbent assay (ELISA) and was significantly increased under an inflammatory condition when leukocytes were included in the PRP. Fibroblasts and osteoblasts treated with L-PRP, under an inflammatory situation, underwent a greater activation of NFĸB pathway, proliferated significantly less and secreted a higher concentration of pro-inflammatory cytokines. These cellular events were assessed through Western blot and fluorimetric and ELISA methods, respectively. Therefore, the inclusion of leukocytes induced significantly higher pro-inflammatory conditions.

## Introduction

Over the last few years, translational medicine has emerged as a new trend in medical practice, which aims to promote the rapid clinical translation of a wide range of therapeutic options and to receive the feedback of basic research results [1]. One of the hottest topics in translation medicine deals with those therapies focused on enhancing and promoting tissue repair and regeneration [2]. Repair, restoration and healing of lost tissue are unique among clinical treatments due to the large number of patients suffering from tissue damage and injuries. Progress in this field is pertinent as it may help to reduce the burden on the world’s health systems and address the need of tissue replacement [3].

The use of patient-derived formulations and therapeutics is gaining the attention of the scientific community. Human plasma and especially human platelets contain a wide range of proteins and growth factors that have been demonstrated to promote tissue repair and regeneration in many different injured tissues [4–14]. In addition, the coagulation of plasma leads to the formation of a three-dimensional fibrin matrix that can provide transient space for the key tissue-forming cells at the same time that acts as a controlled protein delivery system [2, 13]. These and other advances have promoted the research and use of platelet rich plasma products as a biomolecules delivering system [15] for wound healing, tissue regeneration and tissue engineering [16].

There are however, several concerns related to the use of platelet rich plasma products that need to be addressed. First, how these preparations exert their effects in inflammatory conditions. To our knowledge, most of the studies evaluating the biological potential of platelet rich plasma products do not mimic tissue inflammatory conditions in which most of these therapies are used. Second, how the particular protein and cell composition of these products can affect their final therapeutic outcomes. In fact, while some platelet rich plasma products contain only platelet-enriched plasma, others concentrate leukocytes, red cells and other pro-inflammatory cells. As a consequence, the composition and biological properties of these products might be potentially different. It has been demonstrated that leukocyte concentration is negatively correlated with matrix gene expression and positively correlated with catabolic gene expression in tendon and ligament tissues [17]. Moreover, white blood cells (WBC) contain numerous pro-inflammatory interleukins and extracellular matrix degrading enzymes [18]. Third, only few studies have determined the role and properties of the fibrin scaffold formed with each type of platelet rich plasma product.

In the present study, we have carried out a complete evaluation of how including leukocytes (L-PRP) or not in plasma rich in growth factors (PRGF), a well-characterized platelet rich plasma therapy, may alter both protein and cytokine release and fibrin scaffold properties. Leukocyte containing and leukocyte free PRGFs were prepared and evaluated in non-inflammatory and inflammatory conditions that mimic a well-known chronic inflammatory disease such as periodontitis. The latter is caused by *Porphyromonas gingivalis* [19] and affects periodontal tissue due to the release of proteolytic enzymes [20, 21].

## Materials and Methods

The study was performed following the principles of the Declaration of Helsinki, as revised in 2008 and after approval from the Foundation Eduardo Anitua Institutional Review Board.

### Preparation of PRP (PRGF) products

Blood from a total of 6 patients was collected into 9-mL tubes with 3.8% (wt/v) sodium citrate, after written informed consent was provided (approved by our Institutional Review Board). The following products were generated immediately from the collected blood: plasma rich in growth factors (PRGF) and Leukocyte-PRP (L-PRP). PRGF was prepared according to manufacturer’s instructions (Endoret Dentistry, BTI Biotechnology Institute, S.L., Miñano, Álava, Spain). Briefly, samples were centrifuged at 580 *g* for 8 min at room temperature. For each tube, the 2 ml of plasma just above the buffy coat was collected in each donor. Leukocyte-PRP was prepared by the addition of the buffy coat into the platelet richest fraction. Platelets and leukocytes counts were performed on samples of blood, PRGF and L-PRP with a hematology analyzer (Micros 60, Horiba ABX, Montpelier, France).

### Structure of fibrin net from both PRP products

The different fibrin scaffolds were prepared by activating different volumes of PRGF and L-PRP with calcium chloride (Endoret Dentistry) at 37°C for 1 hour (50 μL of calcium chloride for each mL of PRGF/L-PRP). After clot formation, the morphological analysis of PRGF and L-PRP fibrin scaffolds was performed with scanning electron microscopy (SEM). Briefly, the clots were rinsed with phosphate-buffered saline and fixed with 2% glutaraldehyde in 0.1 M cacodylate buffer for 4 h and washed in cacodylate-sucrose buffer. Then, the samples were post-fixed with osmium tetroxide for 1 h, washed again and dehydrated with serial concentrations of ethanol. Samples were critical point-dried (Tousimis Autosamdri 814), sputter-coated with 5 nm gold (Edwards E306A) and subsequently examined in a scanning electron microscope (Hitachi S-4800) [22].

### Mechanical properties of PRGF and L-PRP fibrin scaffolds

The mechanical properties of PRGF and L-PRP fibrin scaffolds were evaluated. Two mL of each formulation and 100 μL of calcium chloride were added to the devices. Once the clot was formed, the devices were held on the mechanical testing station (858 Mini Bionix II, MTS, USA) with a loading cell of 10N, and de-moulded, leaving the fibrins ready to perform the assays. The mechanical testing station stretched the fibrin scaffolds at a constant speed of 2mm/s. Maximum elongation at failure was determined.

### Culture of PRGF and L-PRP on inflammatory and non-inflammatory conditions

Two mL of PRGF or L-PRP of each of the six donors were added to each well of a 6-well cell culture plate and incubated with 100 μL of calcium chloride for 20–30 min at 37°C until clot formation. Thereafter, half of the scaffolds of both PRGF and L-PRP were incubated with 2 mL of Dulbecco’s modified Eagle’s medium (DMEM)/F12 (Gibco-Invitrogen, Grand Island, NY, USA) in the absence (normal condition) or presence (inflammatory condition) of 10 μg/ml of LPS from *Porphyromonas gingivalis* (*P*.*g*.-derived LPS) (InvivoGen, San Diego, CA, USA) at 37°C with 5% CO_2_ for 72h. The remaining scaffolds were incubated with 2 mL of osteoblast basal medium (ObM) (Sciencell Research Laboratories, Carlsbad, CA, USA), also under both normal and inflammatory conditions. After that, the conditioned media by the scaffolds was collected and centrifuged at 460 g for 10 min at room temperature. The supernatant was stored at -80°C for assay of protein levels.

### Quantification of cytokines and growth factors by ELISA

The levels of interleukin 1 beta (IL-1β), interleukin 8 (IL-8) (Invitrogen), tumor necrosis factor-α (TNF-α, interleukin 6 (IL-6), vascular endothelial growth factor (VEGF), platelet-derived growth factor AB (PDGF-AB) and angiopoietin-1 (R&D Systems, Minneapolis, MN, USA) on the conditioned media of both PRGF and L-PRP clots under both normal and inflammatory conditions, were determined by enzyme-linked immunosorbent assay (ELISA), according to the manufacturer’s protocol.

### MMP-1 activity assay

In order to determine the MMP-1 activity on the conditioned media of both PRGF and L-PRP clots under both study conditions, a MMP-1 colorimetric drug discovery kit (Enzo Life Sciences, Lausen, Switzerland) was used with some modifications to the manufacturer’s protocol. A standard curve for MMP-1 was performed. The enzyme activity was determined 1 minute after the reaction was started.

### Cell treatment with PRGF and L-PRP

Primary human gingival fibroblasts and primary human alveolar osteoblasts, obtained from patients during routine surgical procedures and dental implant surgery, respectively, were isolated as previously described [23, 24] and were used to assess the effect of both PRGF and L-PRP releasates under inflammatory conditions. Written informed consent from those patients and approval by the Foundation Eduardo Anitua institutional review board were obtained.

Fibroblasts were routinely cultured in DMEM/F12 supplemented with 2 mM glutamine and 50 μg/mL gentamicin (Sigma-Aldrich, St. Louis, MO). Osteoblasts were cultured in ObM supplemented with 50 μg/mL gentamicin. Both cultured media were supplemented with 15% fetal bovine serum FBS (Biochrom AG, Leonorenstr, Berlin, Germany).

**Fig 1:**
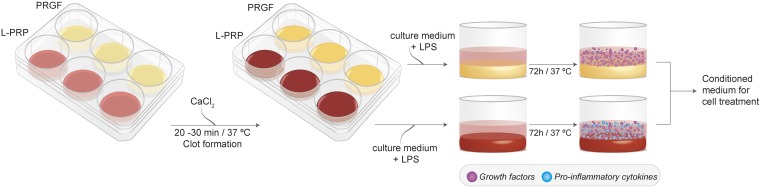
Illustrative representation of the cell treatment experimental procedure with PRGF and L-PRP releasates under inflammatory conditions.

PRGF and L-PRP scaffolds from one donor were stimulated with 10 μg/mL of *P*.*g*.-derived LPS for 72 h, as previously described (Fig 1). The molecule releasates from the scaffolds incubated with LPS will be identified as PRGF+LPS and L-PRP+LPS.

The cell incubation experiments were performed as follows: Cells were pretreated with 10 μg/ml of *P*.*g*.-derived LPS in the appropriate culture medium for 72 hours. The pretreated cells were then cultured with the corresponding culture medium and 10 μg/ml of *P*.*g*.-derived LPS supplemented with either 70% PRGF+LPS or 70% of L-PRP+LPS. The treatment time was 24 h for Western blot analysis, 72 h for proliferation assay, and 24h and 72h for cytokine secretion assay.

### Western blot analysis

After treatment, both fibroblasts and osteoblasts were washed with PBS and lysed with mammalian protein extraction reagent supplemented with protease and phosphatase inhibitors (Pierce Biotechnology, Bonn, Germany). Lysates were clarified by centrifugation at 14000 g for 10 min, and the supernatants were then collected. Cell lysates were concentrated using centrifugal filters (Amicon ultra-0.5 (3k), Chemicon-Millipore, Billerica, MA, USA) and the protein concentration was determined with the BCA assay (Pierce Biotechnology). TGX stain-free gels were used for protein electrophoresis (Bio-Rad Laboratories, Munich, Germany). The membranes were blocked using 5% non-fat dry milk in tris (hydroxymethyl) aminomethane-buffered saline containing 0.1% polysorbate surfactant (TBST) for 1 hour at room temperature. Then the membranes were incubated overnight at 4°C with the corresponding primary antibodies, nuclear factor-ĸB p65 (NF-ĸB p65), phosphorylated nuclear factor-ĸB p65 (pNF-ĸB p65) and inhibitor-ĸBα (IĸBα) (Cell signaling Technology, USA). Membranes were then washed several times with TBST and incubated with horseradish peroxidase-conjugated goat anti-rabbit and anti-mouse secondary antibodies (Cell signaling Technology) accordingly, for 1 hour. The blots were washed again and developed by chemiluminescence with substrate Immun-Star HRP substrate, (Bio-Rad Laboratories) using an image analyzer (Chemidoc image analyzer, Bio-Rad Laboratories). The Stain-Free technology was used as loading control method [25].

### Cell Proliferation assay

Cell proliferation was evaluated by Cyquant cell proliferation assay (Molecular Probes-Invitrogen, Grand Island, NY, USA). Briefly, the treatments were removed and the wells were washed carefully with phosphate-buffered saline (PBS). Then, the microplates were frozen at -80°C until assayed, to allow cell lysis. After thawing the plates at room temperature, wells were incubated with RNase A (1.35 Ku/ml) diluted in cell lysis buffer for 1 hour at room temperature. Then, 2x GR dye/cell-lysis buffer was added to each sample well, mixed gently and incubated for 5 minutes at room temperature, protected from light. Sample fluorescence was measured with a fluorescence microplate reader (Twinkle LB 970, Berthold Technologies, Bad Wildbad, Germany). A DNA standard curve was included in each assay.

### Cell-stimulated cytokine expression

ELISA was performed to analyze the concentration of pro-inflammatory cytokines secreted by human gingival fibroblasts and alveolar osteoblasts after treatment with the conditioned media from both PRGF and L-PRP. Briefly, the conditioned media was collected and levels of IL-1β, TNF-α IL-6 and IL-8 were measured by ELISA kits (R&D Systems). The concentrations of these proteins were determined according to the manufacturer’s instructions.

### Statistical Analysis

The results were presented as mean ± standard deviations. Single-factor analysis of variance (ANOVA) and Kruskal-Wallis test for independent samples was used, as appropriate, to test the differences among groups. P < 0.05 was considered to indicate statistical significance.

## Results

### Composition and structure of fibrin scaffolds from PRGF and L-PRP

**Fig 2:**
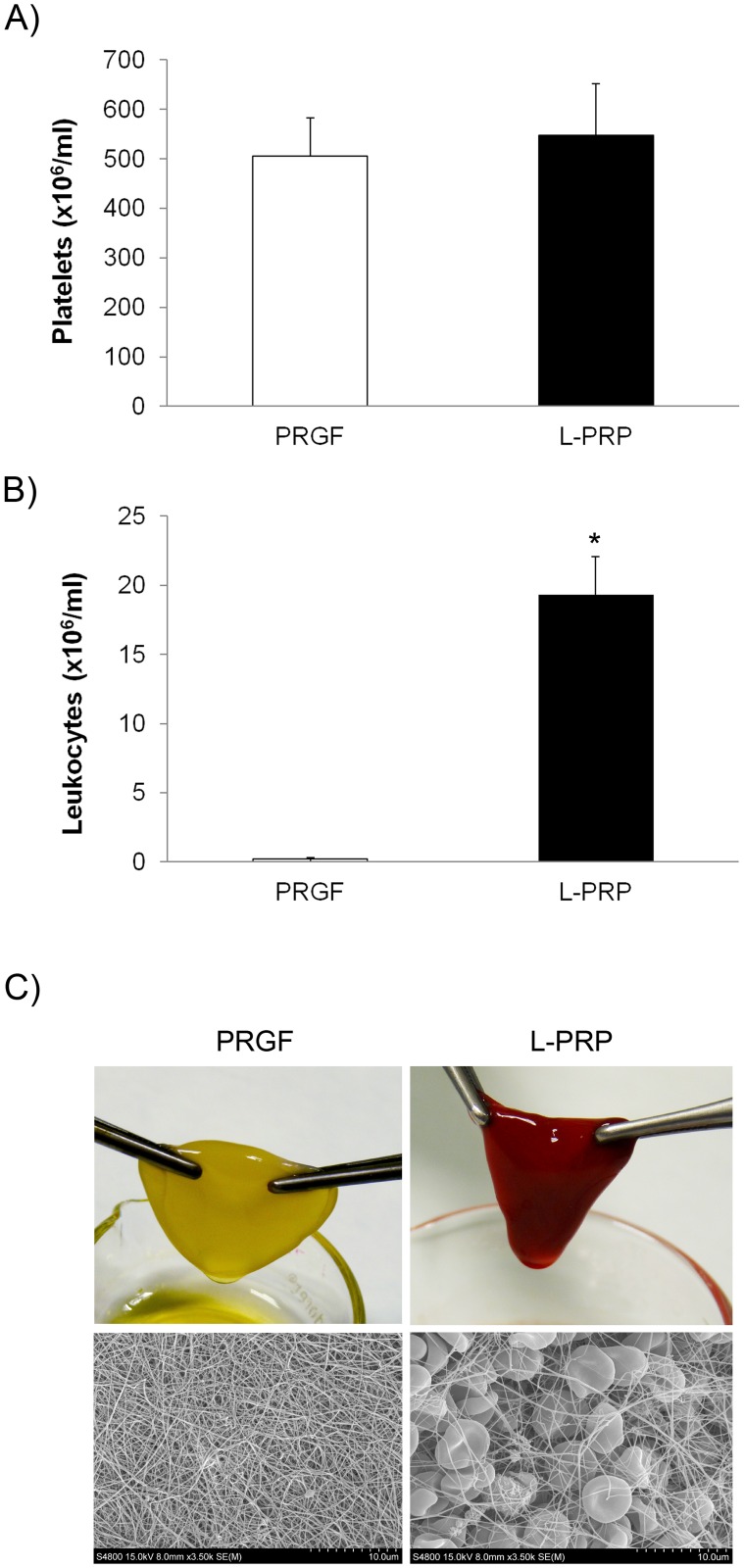
Composition and structure of fibrin net from both PRP products. (A) Concentration of platelets in PRGF and L-PRP preparations. (B) Leukocyte content in PRGF and L-PRP preparations. (C) Macroscopic appearance and ultraestructural composition of PRGF and L-PRP scaffolds.

Cell content of PRGF and L-PRP preparations was measured (Fig 2A). No statistically significant differences were found in platelet counting. However, leukocyte concentration in L-PRP was 3.1 ± 0.3-fold higher than in blood while almost no white cell was detected in PRGF (0.2 ± 0.1 x10^3^/μl) (Fig 2B).

The fibrin scaffolds obtained with each type of platelet rich plasma showed different appearance and consistency when handled. PRGF fibrin scaffold was yellowish and easier to handle than the reddish one prepared from L-PRP (Fig 2C). In fact, the latter was easily broken after manipulation. Ultra structural analysis of both fibrin scaffolds showed the alteration of the fibrillar cross-linking due to the presence of cellular elements including leukocytes and erythrocytes in the L-PRP (Fig 2C).

### Characterization of the PRGF and L-PRP-scaffold releasates

**Fig 3:**
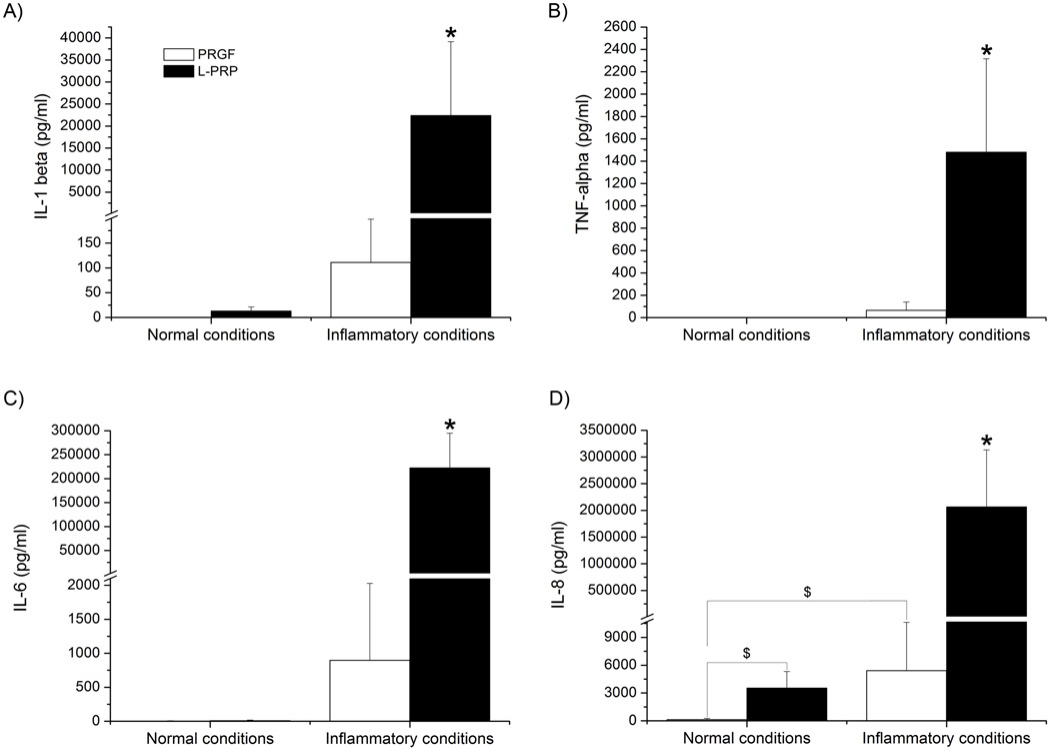
Characterization of the PRP scaffold releasates. Concentration of different cytokines, (A) IL1-beta, (B) TNF-alpha, (C) IL-6 and (D) IL-8 in the PRGF and L-PRP releasate under both normal and inflammatory conditions. *Statistically significant differences respect to the other treatments (p < 0.05). $Statistically significant differences respect to the PRGF treatment under normal conditions (p < 0.05).

The different fibrin scaffolds were cultured at 37°C either in non-inflammatory (only culture medium: normal conditions) or inflammatory (LPS-containing) conditions. After 72 hours, several growth factors (GFs) involved in different phases of the regeneration process and the most relevant interleukins and pro-inflammatory molecules were measured. Under normal conditions, the IL-1β concentration in the PRGF releasate was 0.0 ± 0.4 pg/ml while it was higher in the L-PRP releasate (13 ± 8.5 pg/mL). However, under inflammatory conditions, these difference increased drastically, being the IL-1β concentration in the PRGF releasate of 111 ± 87 pg/mL and 22390 ± 16745 pg/mL in the L-PRP supernatant (Fig 3A). The presence of TNF-α and IL-6 cytokines in the PRGF and L-PRP releasates was almost non-detectable in normal conditions. However, once again, those levels were significantly higher in the case of L-PRP releasate under inflammatory conditions. TNF-α and IL-6 levels in PRGF were 66 ± 74 pg/mL and 896 ± 1131 pg/mL respectively, while in the case of L-PRP they were 1481 ± 835 pg/mL and 222100 ± 72878 pg/mL respectively (Fig 3B and 3C). Furthermore, a statistically significant higher IL-8 delivery from L-PRP scaffolds was measured in both analyzed conditions with respect to the PRGF scaffold releasates (129 ± 139 pg/mL *vs* 3535 ± 1791 pg/mL and 5415 ± 5207 pg/ml *vs* 2065000 ± 1067263 pg/ml, for PRGF and L-PRP under normal and inflammatory conditions, respectively) (Fig 3D).

**Fig 4:**
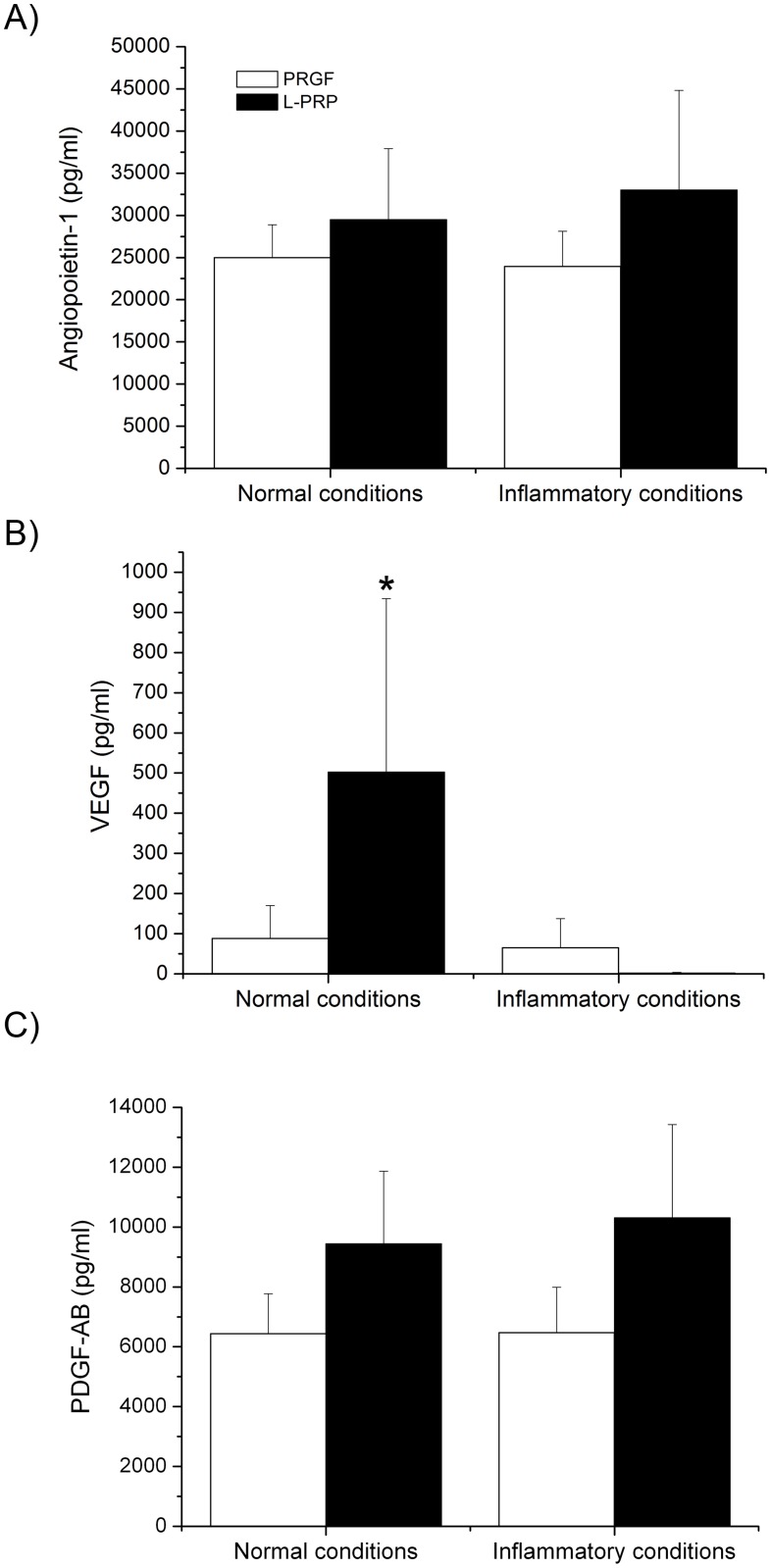
Characterization of the PRP scaffold releasate. Determination of different proteins concentration. (A) Angiopoietin-1 (B) VEGF and (C) PDGF-AB in the PRGF and L-PRP releasate under both normal and inflammatory conditions. *Statistically significant differences respect to the other treatments (p < 0.05).

Release of PDGF and two additional proteins with important roles in angiogenesis, Angiopoietin-1 and VEGF, were analyzed in the different releasates. In the case of PDGF-AB and angiopoietin-1, no statistically significant differences were found between PRGF and L-PRP releasates either in normal or inflammatory condition (Fig 4A and 4C). Nevertheless, VEGF delivery from L-PRP scaffold was significantly higher than the PRGF one, but only in the absence of inflammation (88 ± 82 pg/ml *vs* 502 ± 432 pg/ml). Interestingly, under inflammatory conditions, no VEGF was detectable in the L-PRP releasate whereas VEGF levels were maintained in the case of PRGF releasates (Fig 4B).

### Analysis of the integrity and the mechanical properties of the fibrin scaffolds

**Fig 5:**
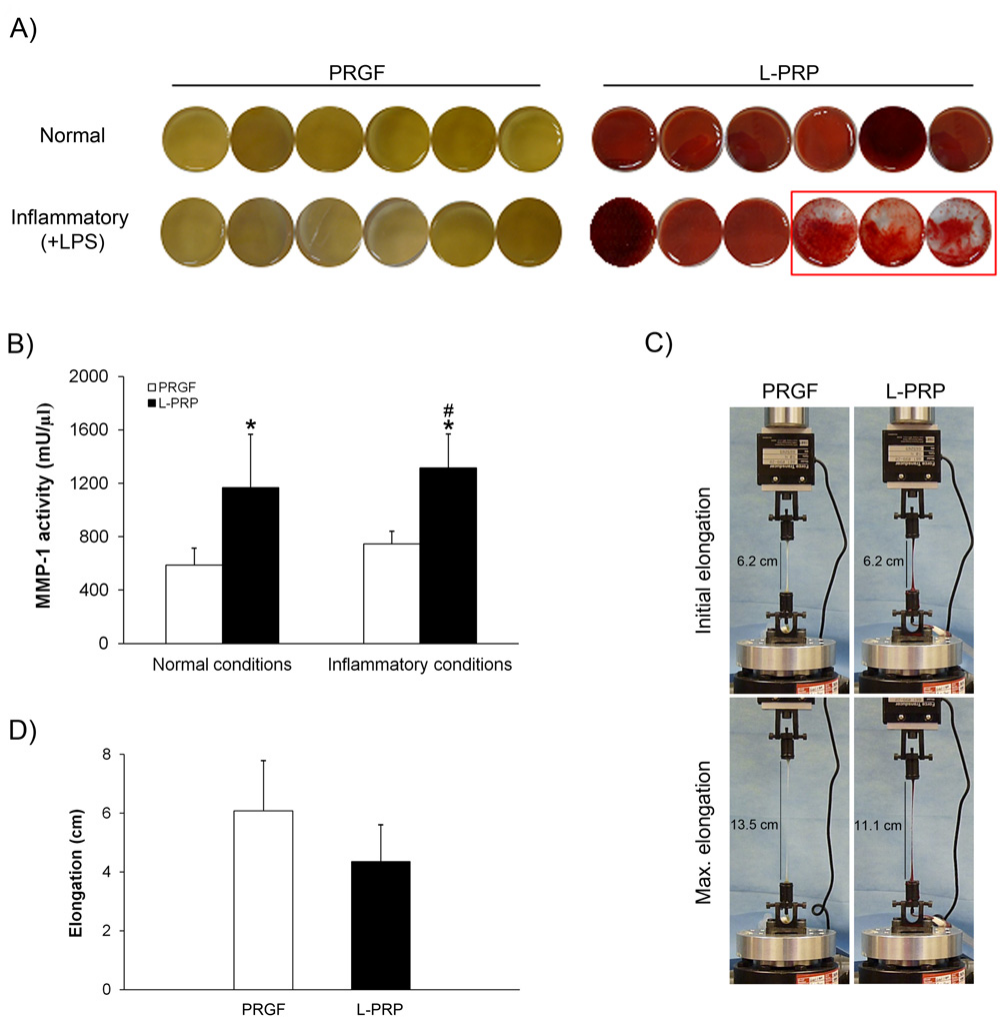
Analysis of the integrity and the mechanical properties of PRP scaffolds. (A) Comparison of PRP scaffolds appearance after 72 hours incubations under normal and inflammatory conditions. In half of the L-PRP matrix a great degradation was observed. (B) MMP-1 activity in PRGF and L-PRP releasates was determined under both normal and inflammatory conditions. (One U = 100 pmol/min at 37°C, 100 μM thiopeptolide). (C) Mechanical testing station where the maximum elongation at failure of the PRP scaffolds was determined. (D) Maximum elongation of PRP scaffolds. *Statistically significant differences between L-PRP and PRGF under normal conditions and between L-PRP and PRGF under inflammatory conditions (p < 0.05).^#^ Statistically significant differences respect to the PRGF treatment under normal conditions (p < 0.05).

PRGF and L-PRP-derived fibrin scaffolds from each of the six donors cultured with culture medium were maintained in the absence (normal condition) or presence (inflammatory condition) of LPS for 72h. PRGF fibrin scaffold preserved its stability under both normal and inflammatory conditions, while in the case of L-PRP, the fibrin scaffold were altered due to the presence of LPS (Fig 5A). In fact, half of the L-PRP fibrin scaffolds which were incubated with LPS appeared highly deteriorated after 72 hour of treatment, as it is shown in Fig 5A.

MMP-1 activity was measured in the different releasates under normal and inflammatory conditions. A statistically significant higher enzyme activity was detected in the L-PRP scaffold conditioned medium under both study conditions (586 ± 128 mU/μl respect to 1168 ± 401 mU/μl for PRGF- and L-PRP-releasates under normal conditions and 745 ± 96 mU/μl respect to 1315 ± 257 mU/μl for PRGF- and L-PRP-releasates under inflammatory conditions). The detected enzyme activity in the L-PRP-releasates under inflammatory conditions was also statistically superior than the one detected in the PRGF-releasates under normal conditions (Fig 5B).

In addition, the mechanical properties of the different fibrin scaffolds were determined. For this purpose, the maximum elongation at failure was determined after stretching fibrins at a constant speed of 2mm/s. The maximum elongation achieved with the PRGF scaffolds was higher than with L-PRP ones (6.1 ± 1.7 cm *vs* 4.4 ± 1.3 cm) (Fig 5C and 5D).

### Evaluation of cell inflammatory NFĸB pathway

**Fig 6:**
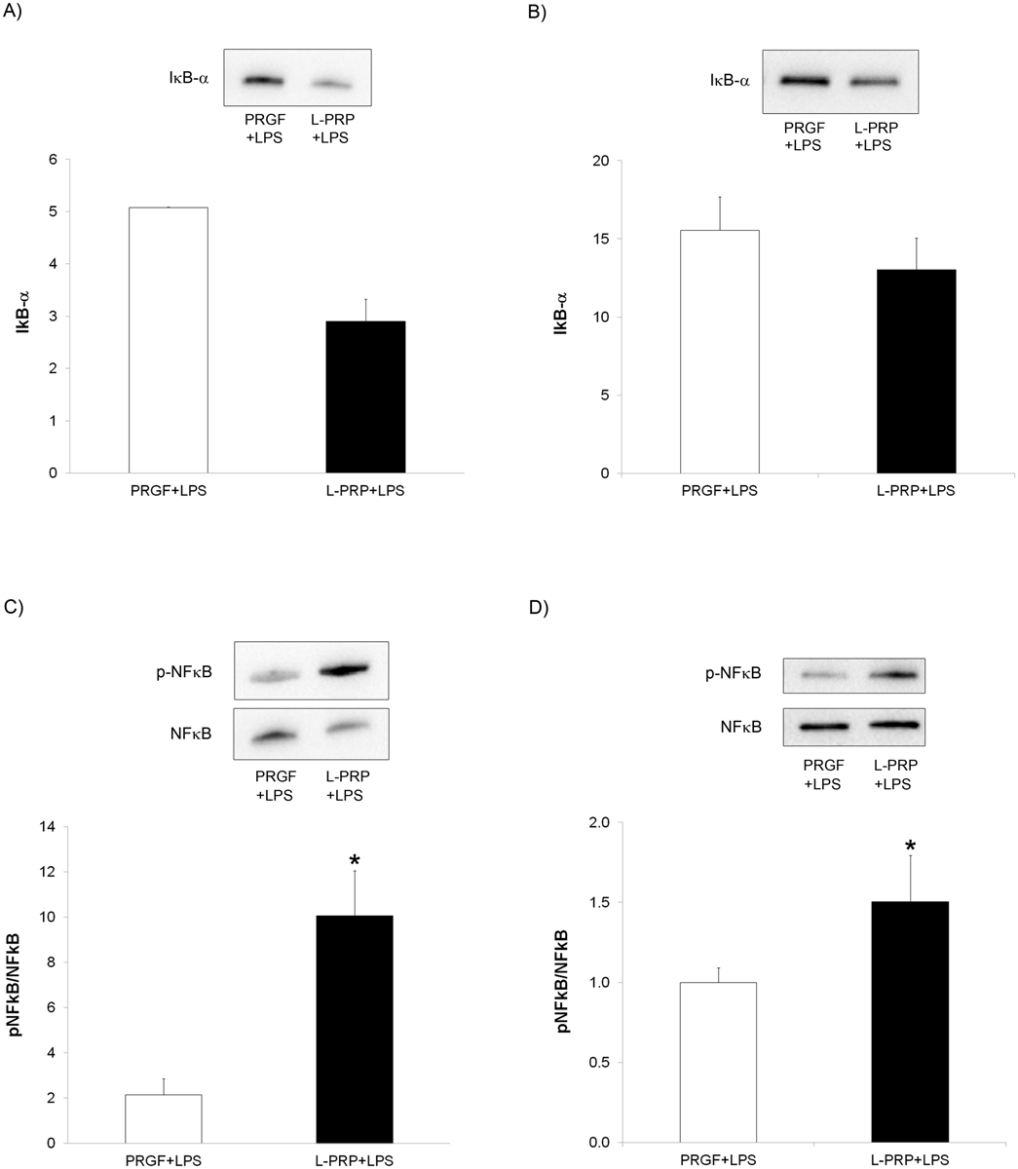
Analysis of the inflammatory mediators’ expression by western blot. Cells were treated with PRGF+LPS and L-PRP+LPS releasates for 24 hours under inflammatory conditions and IĸB-α and p-NFĸB/NFĸB ratio were determined. (A) IĸB-α expression in gingival fibroblasts. (B) IĸB-α expression in alveolar osteoblasts. (C) p-NFĸB/NFĸB determination in gingival fibroblasts. (D) p-NFĸB/NFĸB determination in alveolar osteoblasts. *Statistically significant differences respect to the PRGF+LPS treatment (p < 0.05).

Primary human gingival fibroblasts and alveolar osteoblasts were treated with conditioned media by PRGF and L-PRP scaffolds under inflammatory conditions for 24 hours. After extracting and concentrating the protein content of the treated cells, IĸB-α, p-NFĸB and NFĸB synthesis were quantified by western blotting technique. In both of the two phenotypes included in the study, a decrease in IĸB-α expression was found when cells were incubated with the L-PRP releasate compared with the ones incubated with PRGF releasates (5.1 ± 0.0 *vs* 2.9 ± 0.4 and 15.6 ± 2.1 vs 13.7 ± 2.3 for PRGF and L-PRP in gingival and bone cells, respectively) (Fig 6A and 6B). On the contrary, a statistically significant increase in the p-NFĸB/NFĸB ratio was observed when cells were treated with L-PRP+LPS releasate when compared with PRGF+LPS treatment (2.1 ± 0.7 *vs* 10.0 ± 2.0 and 1.0 ± 0.1 *vs* 1.5 ± 0.3 for PRGF and L-PRP and in gingival and osteoblast cells respectively) (Fig 6C and 6D).

### Cell proliferation assay

**Fig 7:**
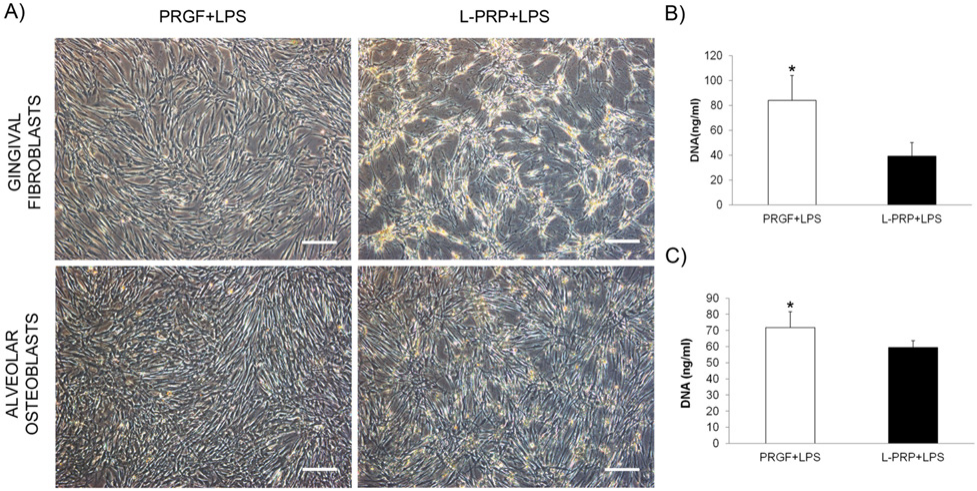
Effect of the releasates of PRGF+LPS and L-PRP+LPS on the proliferation of fibroblasts and osteoblast cells. (A) Morphological alterations in cultures due to 72 hour treatment with PRP releasates under inflammatory conditions. Scale bar: 300 μm. (B) Gingival fibroblast proliferation after treatment. (C) DNA quantification of alveolar osteoblast proliferation after treatment. *Statistically significant differences respect to the L-PRP+LPS treatment (p < 0.05).

Fibroblasts and osteoblasts were incubated with PRGF+LPS and L-PRP+LPS releasates for 72 hours and in LPS presence. Some morphological changes were observed after the treatment with L-PRP+LPS scaffold releasate, especially in the case of gingival fibroblasts. As it shown in Fig 7A, cells laid out in clusters and became brighter and with a thinner cell body. In addition, a statistically significant decrease in DNA concentration was detected in gingival fibroblast and osteoblast cultures incubated with L-PRP+LPS conditioned medium under inflammatory conditions compared to the ones detected after PRGF+LPS releasate treatment (84 ± 20 ng/ml *vs* 39 ± 11 ng/ml and 72 ± 10 ng/ml *vs* 60 ± 4 ng/ml for PRGF and L-PRP in gingival and bone cell cultures, respectively) (Fig 7B and 7C).

### Cell-derived cytokine expression

**Fig 8:**
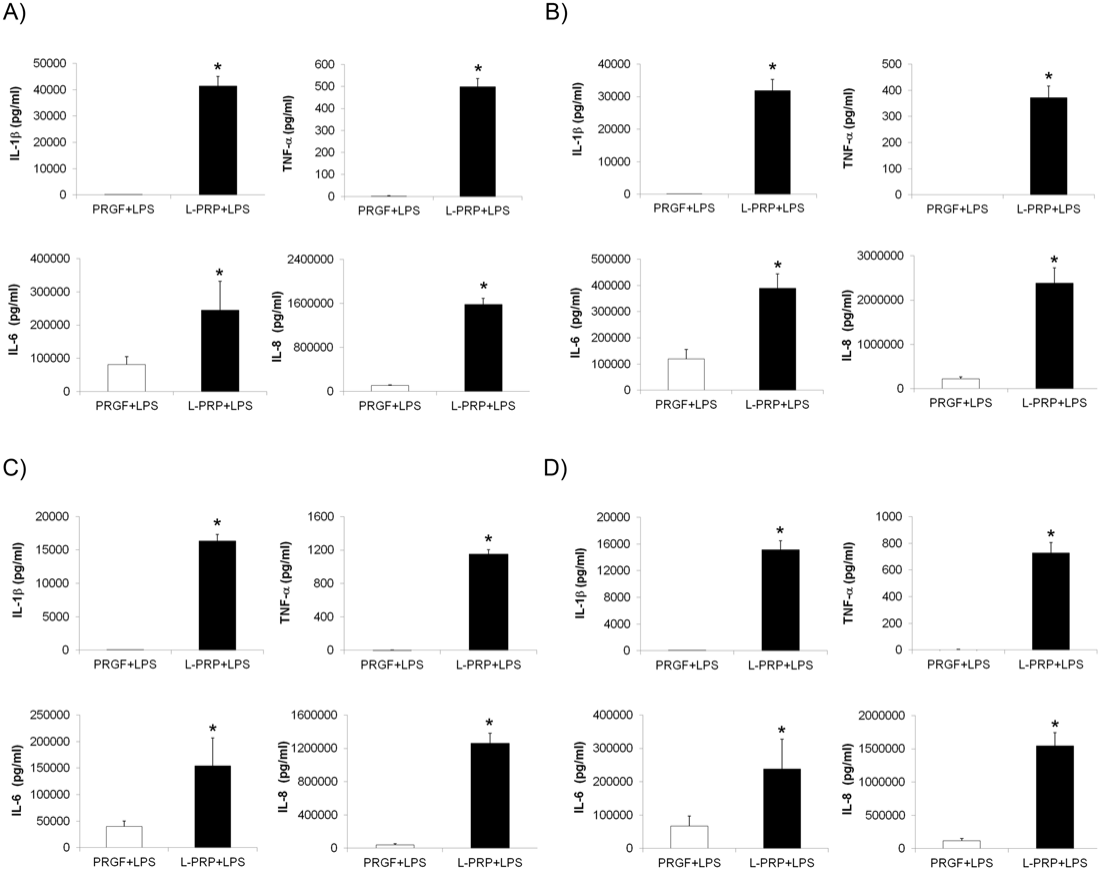
Pro-inflammatory cytokine synthesis due to cell treatment with PRP releasates. Gingival and bone cells were incubated with PRGF+LPS and L-PRP+LPS releasates under inflammatory conditions for 24 and 72 hours. IL-1β, TNF-α, IL-6 and IL-8 synthesis was determined by ELISA techniques. (A) Treatment of gingival fibroblast during 24 hours. (B) Gingival fibroblast response to 72 hour treatment. (C) Treatment of alveolar osteoblasts during 24 hours. (D) Bone cells response to 72 hour treatment. *Statistically significant differences respect to the PRGF+LPS treatment (p < 0.05).

After treating gingival and bone cells for 24 and 72 hours with PRGF+LPS and L-PRP+LPS, the conditioned media was collected and the levels of IL-1β, TNF-α, IL-6 and IL-8 were measured. The concentration of all the pro-inflammatory cytokines was significantly enhanced after treating the cells with L-PRP+LPS releasate (Fig 8).

As it is shown in Fig 8A (24 h treatment), gingival fibroblasts stimulated with PRGF released 6 ± 2 pg/mL of IL-1β while they released up to 41492 ± 3629 pg/mL when stimulated with L-PRP. Gingival cell expression of TNF-α was also significantly increased with L-PRP (500 ± 36 pg/mL) compared to PRGF (0 ± 3 pg/mL). A similar trend was observed for IL-6 and IL-8 expression. Cell culture with L-PRP significantly increased the amount of the proteins (244833 ± 87279 pg/mL for IL-6 and 1583333 ± 111430 pg/mL for IL-8) compared to those obtained with PRGF (80992 ± 23878 pg/mL and 110492 ± 7569 pg/mL for IL-6 and IL-8, respectively). A similar trend was observed after treating the cells for 72 h with PRGF or L-PRP (Fig 8B).

In a second set of experiments, the effects of the conditioned media on primary osteoblasts cells were evaluated (Fig 8C and 8D). Culturing the cells with the L-PRP media provoked the dramatic increase of the expression of the four cytokines. Interestingly, the use of a leukocyte containing PRP enhanced osteoblasts-derived release of IL-1β more than 700 times (Fig 8C). This striking increase in the synthesis of pro-inflammatory cytokines due to the leukocyte inclusion in the PRP-scaffold was also observed for the remaining three proteins. Osteoblast cell expressions of TNF-α (1152 ± 53 pg/mL), IL-6 (154333 ± 52495 pg/mL) and IL-8 (1259667 ± 124582 pg/mL) were significantly higher than those observed after PRGF treatment TNF-α (-3 ± 9 pg/mL), IL-6 (40050 ± 10300 pg/mL) and IL-8 (41056 ± 14340 pg/mL). A similar trend was observed after treating the cells for 72 h with PRGF or L-PRP (Fig 8D).

## Discussion

Periodontal disease is the most prevalent chronic inflammatory condition in humans. It is characterized by the destruction of the tooth-supporting structures, namely, soft tissue, alveolar bone, periodontal ligament and cementum [26–28]. *Porphyromonas gingivalis* is a gram-negative anaerobic bacterium that is considered to be the major etiologic agent of periodontitis. Lipopolysaccharide, a major component of the outer membrane of the gram-negative bacteria, is known to be the major virulent factor in periodontitis, as it is a potent stimulator of inflammatory mediators [26, 29, 30].

In the last few decades, there is growing interest in developing new cost-effective therapeutic alternatives for tissue repair and regeneration. Assuming the scarcity of human organs and tissues, there is interest in promoting new approaches that help to restore tissue homeostasis after inflammation and injury. The field of platelet rich plasma, that is, the use of patient’s own proteins, growth factors and biomaterials is gaining much interest not only due to its autologous origin, but also because it is a not expensive approach with a clear translation pathway to the clinics.

In the present paper, we have addressed one of the most important concerns about PRPs; how the composition of leukocytes may impact on the final biological and mechanical properties, which are key for final therapeutic outcomes of the approach. In addition, to our knowledge this is one of the few studies that investigates the effect of PRP composition under normal and inflammatory conditions.

According to our results, the different composition of PRPs containing leukocytes affects negatively the mechanical properties of their fibrin scaffolds and stimulates a more pro-inflammatory environment that is directly related with an increased cell-inflammatory condition and a reduced cell proliferation response. These effects may delay or impede a correct tissue repair or regeneration process.

The inclusion of leukocytes within platelet rich plasma products has always been a matter of debate. The deleterious effects of leukocytes have been widely described [31–34]. In contrast to platelets, white blood cells are mainly considered to contain and produce inflammatory cytokines.[31, 32, 35, 36] The results obtained in the present study confirm that leukocyte free PRGF is more predictable as its properties (growth factor release and fibrin scaffold integrity) are almost preserved from non-inflammatory to inflammatory conditions [27, 37].

In our experiments, only the IL-8 released was found to be statistically significant different under normal conditions between PRGF and L-PRP scaffolds, being much higher in the case of L-PRP (Fig 3). However, under inflammatory conditions, the levels of IL-1β, IL-6, IL-8 and TNF-α released by L-PRP fibrin scaffolds were statistically much higher with respect to PRGF (Fig 3). Moreover, in the case of PRGF, no differences were found in protein release between normal or inflammatory conditions, except for IL-8.

Although cytokines play an important role in infection and inflammation, excessive cytokine expression may lead to tissue destruction [38, 39]. Inflammatory cytokines such as IL-6 and IL-8 are believed to be the main pathological mediators in periodontal diseases. IL-6 may activate osteoclasts, promoting bone loss and causing bone resorption. At the same time, this cytokine stimulates matrix metalloproteinases, therefore increasing matrix degradation. IL-8 in turn, may induce neutrophil chemotaxis and activation [40–42]. Meanwhile, TNF-α and IL-1β have also a significant role in bone loss, playing a central role in inflammatory reaction [28, 43]. In fact, IL-1β is an essential pro-inflammatory cytokine that induces the infiltration of inflammatory cells [44].

Apart from the increased enzyme activity of MMP-1 in the L-PRP-releasates under both normal and inflammatory conditions, in the latter, half of the L-PRP fibrin scaffolds were degraded (Fig 5A and 5B). Matrix metalloproteinases (MMPs), a family of proteolytic enzymes, are responsible for degrading the main components of the extracellular matrix. MMP-1 is a collagenase that is involved in extracellular matrix breakdown during periodontitis. The production of this enzyme is stimulated by several growth factors and cytokines such as IL-1β, TNF-α and IL-6 [42, 45].

Several important differences were also observed after determining the release of growth factors from PRGF and L-PRP scaffolds. In normal conditions, the concentration of VEGF released by L-PRP scaffolds was higher than that released by PRGF scaffolds. However, under inflammatory conditions no VEGF was detected in the L-PRP fibrin-conditioned medium. The latter may be a limitation since VEGF is necessary in wound healing causing inflammation as it promotes the early events in angiogenesis [46, 47]. This finding could be explained by a possible VEGF uptake by its soluble receptor (sVEGFR) synthesized by leukocytes [48, 49]. Moreover, similar results were obtained in a previous publication where a VEGF concentration decrease was found in the conditioned medium of L-PRP scaffolds from the third day of assay [50]. Fibrin is the biological transient scaffold that it is firstly formed at injury’s sites and it is commonly used in tissue engineering. The mechanical properties and biodegradation of fibrin scaffolds are essential for their role as a provision matrix in tissue regeneration approaches and as a protein delivery system [2, 51–53]. Viscoelastic properties largely depend on fibrin clot structure. Here, we have reported that the cell composition of L-PRP alters the clot structure and decreases the maximum elongation of the scaffold. Higher elongations indicate a greater elasticity which involves, more malleable, easier to handle and stronger fibrins to fulfill the required function as a biomaterial that can be used as a specific shape scaffold in tissue engineering [51]. PRGF scaffold has already been used in several applications, such as the treatment of ulcers, wound closure, tissue engineering or even combined with others materials [37].

Interestingly, LPS stimulates inflammatory cells, such as neutrophils, macrophages but also resident cells such as fibroblasts and osteoblasts. Therefore, cell responses to the treatment with both types of PRP under inflammatory conditions were also evaluated. Toll-like receptor 4 (TLR4) is the main receptor in the cellular response to LPS. Ligation of these receptors initiates a cascade of events that leads to the activation of transcription factors, including nuclear factor-ĸB (NF-ĸB), that eventually induce the production of pro-inflammatory cytokines [19, 30, 54]. Our findings revealed that the inhibitor-ĸBα (IĸBα expression was increased when gingival fibroblasts and alveolar osteoblasts were cultured with PRGF+LPS under inflammatory conditions (Fig 6). IĸBα is considered to have an anti-inflammatory role as it maintains the NF-ĸB in the cytosol in an inactivated state [55, 56]. These results are consistent with those observed for the p-NFĸB/NFĸB ratio. L-PRP+LPS induced a significantly higher phosphorylation of NF-ĸB p65 in both cell types (Fig 6). NF-ĸB p65 phosphorylation at Ser536 regulates nuclear localization, protein-protein interactions, activation of gene expression and transcriptional activity [56, 57]. Moreover, IL-1β and TNF-α are cytokines that activate the NFĸB pathway [55, 56]. Therefore, the pro-inflammatory environment generated by L-PRP scaffolds may be behind of this increase in the activation of NFĸB after cell treatment with L-PRP+LPS. This pro-inflammatory milieu and the greater activation of NFĸB after treating the cells with L-PRP+LPS had also a negative effect on cell proliferation. Both gingival fibroblasts and alveolar osteoblasts proliferated significantly less when treated with L-PRP+LPS than when treated with PRGF+LPS (Fig 7).

Finally, treatment with L-PRP+LPS also stimulated pro-inflammatory cytokine synthesis in both cell types compared with treatment with PRGF+LPS (Fig 8). This cell response was observed both at 24h and 72h. The excess of those pro-inflammatory proteins can lead to the destruction of the tissue, as previously described. This increase is related to the enhanced activation of NFĸB pathway after the treatment with L-PRP+LPS. In fact, NFĸB activation results in the production of those cytokines, which in turn, can amplify and increase the inflammatory response, thus keeping the NFĸB pathway active, establishing a positive feedback response. In the wake of this activation, the excessive pro-inflammatory cytokine release after L-PRP+LPS incubation may exacerbate the inflammatory response by stimulating the recruitment of new inflammatory cells to the injury site. This microenvironment may be conducive to a non-resolving inflammation that leads to a tissue fibrotic condition [58, 59].

## Conclusions

We have shown that under inflammatory conditions, the different cell composition of L-PRP increases the delivery of pro-inflammatory cytokines, as well as stimulates the resident cells to produce a greater amount of the afore mentioned proteins. The NFĸB pathway is also stimulated in oral cells treated with L-PRP+LPS scaffold releasate under inflammatory conditions. The mechanical properties of the L-PRP scaffolds are poorer and the degradation of these fibrin meshes is promoted under inflammatory conditions. In summary, the composition of L-PRP negatively affects the mechanical properties of the fibrin scaffolds and stimulates a more pro-inflammatory environment that is directly related with an increased cell-inflammatory condition and a reduced cell proliferation response, which ultimately may be detrimental for tissue regeneration.
